# Identification of Cry toxin receptor genes homologs in a *de novo* transcriptome of *Premnotrypes vorax* (Coleoptera: Curculionidae)

**DOI:** 10.1371/journal.pone.0291546

**Published:** 2023-09-14

**Authors:** Luisa-Fernanda Velásquez C., Pablo Emiliano Cantón, Alejandro Sanchez-Flores, Mario Soberón, Alejandra Bravo, Jairo A. Cerón S.

**Affiliations:** 1 Instituto de Biotecnología, Universidad Nacional de Colombia, Bogotá, Colombia; 2 Departamento de Microbiología Molecular, Instituto de Biotecnología, Universidad Nacional Autónoma de México, Cuernavaca, Morelos, México; 3 Unidad Universitaria de Secuenciación Masiva y Bioinformática, Instituto de Biotecnología, Universidad Nacional Autónoma de México, Cuernavaca, Morelos, México; Assam Agricultural University Faculty of Agriculture, INDIA

## Abstract

The white potato worm *Premnotrypes vorax* (Hustache) (Coleoptera: Curculionidae) is one of the most destructive insect pests of potato crops in South America. Like many coleopteran insects, *P*. *vorax* shows low susceptibility to Cry insecticidal proteins produced by the bacterium *Bacillus thuringiensis* (Bt). However, the presence of Cry toxin receptors in the midgut of this this insect has never been studied. The main Cry-binding proteins described in other insect species are cadherin (CAD), aminopeptidase N (APN), alkaline phosphatase (ALP) and ATP-binding cassette (ABC) transporters. In this study, we analyzed and validated a *de novo* assembled transcriptome of Illumina sequencing data to identify and to characterize homologs of Cry toxin receptors. We identified the protein sequences in *P*. *vorax* that show high identity with their orthologous sequences of the Cry toxin binding proteins in other coleopteran larvae such as APN, ALP, CAD and ABC transporter. This study provides preliminary identification of putative receptor genes of Cry proteins that would be useful for future studies involving biocontrol of this important potato crop pest.

## Introduction

Colombia ranks 36^th^ out of 183 countries that produce potatoes (*Solanum tuberosum*) worldwide with more than 60 varieties. The potato is the third most important crop and the average consumption in the country is 60 Kg per person per year [1]. Currently, this crop is a fundamental axis of the country’s economy involving 283 municipalities and more than 110,000 families, mainly in the regions of Boyacá, Cundinamarca, Antioquia and Nariño, which account for more than 85% of the total production of the country. In addition, potato is the crop that generates the greatest number of jobs in cold climate zones (more than 300,000 jobs) since in average 132,161 hectares of potatoes were harvested in the country, having a total national production of 2,751,837 tons [1, 2].

**Fig 1:**
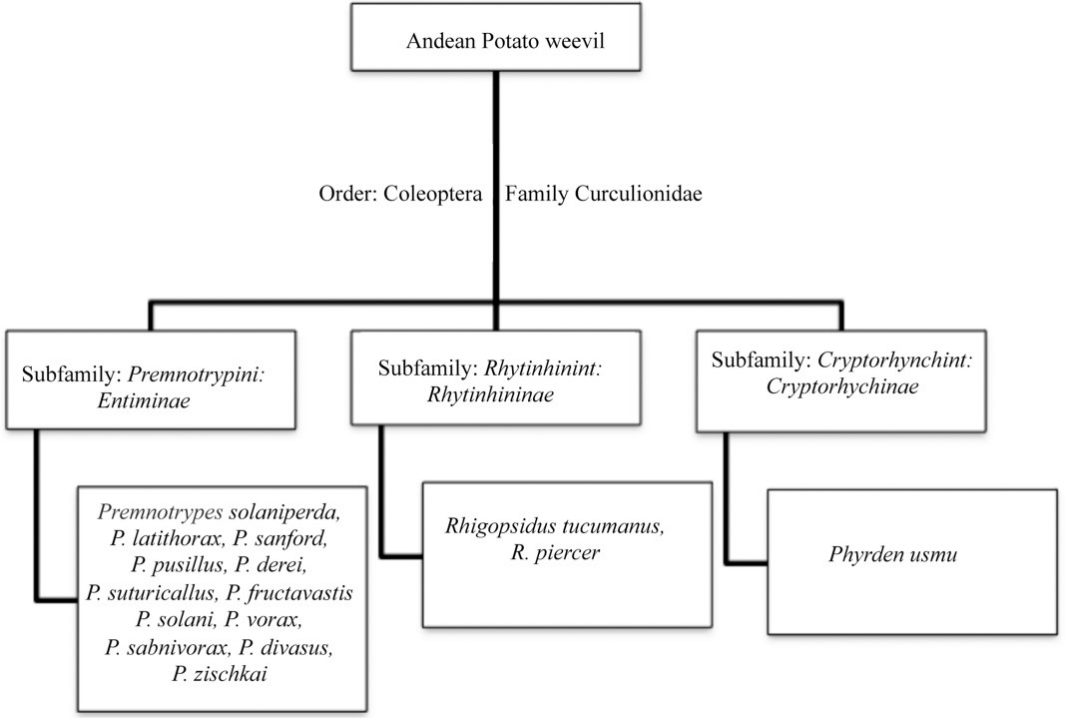
Taxonomy of “Andean potato weevil” complex.

Among the insect pests that affect potato production is *Premnotrypes vorax* (Hustache) (Coleoptera: Curculionidae) also known as white worm of the potato [3]. This is one of the 14 species of potato insect pests that form part of the complex known as “Andean potato weevils” (Fig 1). These insects are mainly distributed in South America, principally in Colombia, Ecuador, Venezuela, and Peru [4]. *P*. *vorax* arrived in Colombia from Ecuador since 1920 at the Nariño region, and from there it was distributed through infected seeds to the most important potato crop regions in Colombia such as Boyacá, Antioquia and Santander [5].

Adult insects of *P*. *vorax* feed on the plant leaves, but the most important damage with economic relevance is caused by the larvae, which feeds on the tubers, making galleries that affect the quality of the product showing external visible damage [6], resulting in up to 90% crop-loses due to the damaged tubers or to the complete crop destruction, especially when the larvae population is high [7]. It was reported that the life cycle of *P*. *vorax* can last up to nine months [8], indicating that it has only one or two generations per year. However, the average lifetime of adults is rather long since it last up to 138 ± 51.7 and 154 ± 50.7 days for males and females, respectively, allowing a very high reproduction rate, since females start oviposition at 14.4 ± 7.8 days after mating and continue up to 91.5 ± 43.0 days, with an average laid eggs of 101.3 ± 78.6 eggs and 9.6 ± 5.5 positions [4]. Historically, the control of this insect by indigenous farmers involves extended crops rotation, special separation between fields, use of plant barriers, and chemical control with highly toxic chemical insecticides such as carbamates, pyrethroids, organophosphates and carbofuran that are applied at the time of potato planting. However, usually these insecticide treatments fail to penetrate deep in the soil, where the potato tuber grows [6, 9]. In Colombia, more than US $22 million are spent each year for spraying insecticides against *P*. *vorax* [10]. In addition, it was reported that in most cases there is mismanagement in the doses and application frequencies of the chemical insecticides, increasing environmental pollution and health risks in both producers and consumers [11, 12].

An ecological alternative for insect pest control, that is compatible with the environment, is the use of bioinsecticides such as the insecticidal proteins produced by the bacterium *Bacillus thuringiensis* (Bt), since they are safe to humans, and completely biodegradable. Bt produce different kinds of insecticidal proteins, where Cry toxins, formerly known as three domain Cry toxins, represent the most widely used at the commercial level worldwide, against Lepidoptera, Diptera and Coleoptera insect pest [13]. Some of the Bt insecticidal proteins with high activity against coleopteran insects have been successfully used to protect different plants against main beetle pests either in insecticidal formulations or expressed them in transgenic crops [14]. For example, the Cry3 proteins have been reported to be active against *Tenebrio molitor* [15] and *Leptinotarsa decemlineata* [16, 17]. The Cry8Ab1 is active against *Holotrichia oblita* [18], while Cry8Ab1 and Cry8Ea1 are toxic against *Holotrichia parallela* [18, 19]. Cry1I, Cry3C, Cry7A, Cry8A, Cry8B, Cry14, Cry18, Cry26, Cry28, Gpp34 (formerly Cry34), Tpp35 (formerly Cry35) have been reported active against *Xanthogaleruca luteola* [20], Cry8Da against *Popillia japonica* [21], Cry3Bb against *Diabrotica virgifera virgifera* [22], Cry7Ab against *Hanosepilachna vigiotioctomaculata* [23] and Cry3Ba against *Tribolium castaneum* [24]. In the case of *P*. *vorax* it was reported that Cry3Aa at 70 μg/mL resulted in 57% mortality of the larvae after 5 days of treatment [25].

The high specificity displayed by Cry toxins resides in the high affinity interactions of Cry insecticidal proteins with their target receptor proteins, which are located on the apical membrane of the insect midgut epithelial cells. Several proteins have been identified in multiple insects as Cry toxin receptors, among them the aminopeptidase-N (APN) and alkaline phosphatase (ALP) proteins that are covalently attached to epithelial membrane by a glycosylphosphatidylinositol (GPI) anchor. Also, some transmembrane proteins such as Cadherin (CAD) and ATP-binding cassette transporter (ABC transporters) subfamilies C2, B1, A2 are recognized as Cry toxin receptors [13]. After ingestion the Cry protoxins, the activated toxins interact with protein receptors that induce conformational changes in the Cry protein that finally insert into the target membrane forming pores, that cause osmotic shock in the midgut cells and death of the insect [13]. The interaction among Cry toxins and receptor proteins that are present in the midgut of insects is a key step in Cry toxicity. High levels of resistance to Cry proteins in different insect species are linked to alterations in Cry-receptors [26]. Insect resistance threatens sustainability of insecticides based on Cry proteins from Bt. For these reasons, the identification of Cry toxin receptors could be useful for stablishing resistance management strategies.

Here we performed a transcriptome study of *P*. *vorax* by RNA-seq technology, which is a powerful technique for description of transcripts and for the measurement of their expression levels. The information presented here will help in establishing genetic basis of putative protein receptors of Cry toxins in *P*. *vorax* for the development of biological strategies for the management of this important insect pest.

## Material and methods

### Total RNA extraction, library preparation and sequencing

A total of 20 *P*. *vorax* larvae collected from potato fields were used to dissect their midgut tissue (20 mg of midgut tissue) and separated in two Eppendorf tubes. The RNA was extracted from these midgut tissues by using the kit Agentcourt RNAdvance Cell v2 (Beckman Coulter) following the manufacturer’s instructions. Samples were kept at -80 ºC until used. Total RNA was dissolved in DEPC treated water and quantified by Nanodrop 1000 (Thermo Scientific) and Qubit 2.0 system. The RNA integrity number (RIN) and concentration of eluted RNA were determined by Bioanalyzer 2100 (Agilent Technologies) system. All samples presented a RIN > 7 (8.5 and 8.8), indicating enough quality and integrity for library preparation. For sequencing, mRNA libraries were prepared from the total RNA using the Illumina TruSeq HT stranded mRNA sample preparation kit following the vendor’s protocol and obtaining an average library fragment size of 500 bp. The mRNA libraries were sequenced by using the HiSeq 2000/2500 platform with a paired end configuration of 300 cycles to generate pair-end reads of 150 bp. General statistics for the sequencing can be found in Table 1.

**Table 1:** General statistics for the sequencing, Trinity assembly, annotation, filtering and completeness evaluation.

Basic statistics	
Sequencing yield (paired reads/Gbases)	383,552,246 / 115.83
Trinity predicted genes	232,969
Trinty reconstructed transcript isoforms	517,235
Total bases of reconstructed transcript isoforms	390.49 Mbases
N50	566 bases
Median transcript length	298 bases
Average transcript length	477.80 bases
Predicted ORFs	870,310
BUSCO completeness (%)	94.1
Filtering and completeness evaluation	
Trinity genes with at least one annotation record	25,631
Trinity transcript isoforms with at least one annotation record	74,984
Total bases of reconstructed transcript isoforms	76.82 Mbases
N50	1,508 bases
Median transcript length	728 bases
Average transcript length	978 bases
Total ORFs after filtering	32,735

### Preprocess, *de novo* assembly, annotation, and mapping

The sequencing quality was analyzed using the FASTQC software using default parameters. Due to the excellent quality, no reads were removed from the dataset and they were assembled using Trinity v2.6.5 [27] under default parameters. The statistics for the generated transcriptome are presented in Table 1. The resulting transcripts were used to predict open reading frames (ORFs) for the probable protein products using TransDecoder v5.3.0 [28] with default parameters. The ORFs were annotated using the Trinotate v3.1.1 [29] to integrate the results from BLASTx, and BLASTp against the UNIPROT databases provided with the software; the HMMSEARCH against the PFAMa database.

### Completeness evaluation and transcriptome filtering

The software BUSCO v3.0.2 [30] was used to evaluate the completeness of the assembled transcriptome. In a nutshell, BUSCO performs a BLASTx search [31] using all transcripts against a database of conserved orthologous proteins from a certain taxonomic clade. For this analysis we used the arthropoda_odb9 database included with the software using the -m transcriptome parameter. The transcriptome filtering was performed based on the annotation results where transcripts having a transcript or ORF with BLASTx, BLASTp or PFAM result, were retained. In order to remove redundancy for further analysis (orthology and phylogenetics), the annotated ORFs were filtered using the CD-HIT program (v4.8.1) [32] using default parameters and a 50% identity threshold for clustering. The transcriptome sequencing data set including the raw and processed data were deposited at NCBI public repositories under the accession number PRJNA506951.

### Orthology and phylogenetic analysis of the *P*. *vorax* transcriptome

An orthology analysis using the ProteinOrtho v6 software (with flags -cpus = 64 -ram = 462144 -p = BLASTp+ -singles) was performed with the *P*. *vorax de novo* transcriptome and the protein databases from other beetle pest species (NCBI id): *T*. *molitor* (GCA_907166875.3); *T*. *castaneum* (GCF_000002335.3); *L*. *decemlineata* (GCF_000500325.1); *Sitophilus oryzae* (GCF_002938485.1); *Dendroctonus ponderosae* (GCF_020466585.1); *Anthonomus grandis grandis* (GCF_022605725.1); *D*. *virgifera virgifera* (GCF_917563875.1). Proteins with a single ortholog in all species, with a predicted protein in *P*. *vorax* from a full transcript, with functional annotation, and a protein length between 70 and 350 amino acids, were selected for the phylogenetic analysis using the RAxML v8.2.12 software with the following parameters: raxmlHPC-PTHREADS-SSE3 -p 070378 -T 32 -m PROTCATIJTTF -# 100 -f a -x 070378. Additionally, UNIGENE annotations were done by using the NCBInr (nonredundant) database and BLASTx software. We performed local BLASTp queries of reported Cry-receptor genes in other insects with BlastStation v 2.75 (TM Software, Inc.), with default parameters, to find homologs in the expressed proteins of the filtered *P*. *vorax* midgut peptides, with the following sequences as queries: for CAD identification the *T*. *molitor* CAH1373803.1 (Gene Bank ID) was used; for ALP the *Manduca sexta* XP_030033710.2 protein; for APN the *Plutella xylostella* NP_001296022.1 protein sequence; for ABCC2 the *Spodoptera frugiperda* AUO38740.1 sequence; for ABCG1 the *P*. *xylostella* AJQ21779.1 sequence; and for the ABCB1 the *D*. *virgifera* XP_050514648.1 protein. The most likely putative Cry-receptor sequence was selected using E-value and identity percentage as criteria. The orthologous proteins previously identified as NCBI nr-annotated UNIGENE in the annotated assemblies of other coleopteran species such as *A*. *grandis*, *D*. *ponderosae*, *D*. *virgifera*, *L*. *decemlineata*, *S*. *oryzae*, *T*. *molitor*, and *T*. *castaneum* were also identified. For each putative *P*. *vorax* Cry receptor, multiple sequence alignments were performed with MUSCLE with all the previously identified orthologs in the selected coleopteran species. These multiple alignments were then used to build neighbor joining trees. Both MUSCLE and phylogeny trees were obtained with MEGA-X v. 10.2.2 [33], with default parameters.

### Additional permits

No additional permits are required, since we are not working with humans or animals that are subject of ethical permits.

## Results and discussion

Previously, the toxicity of Cry3Aa, Cry3Ba, Cry3Bb, Cry3Ca and Cry7Aa proteins was evaluated by means of a bioassay carried out on cubes of potato tubers impregnated with *B*. *thuringiensis* protoxins at 10 μg of protein/cm3 of diet. The data showed that all these proteins display low toxicity towards *P*. *vorax*, lower than 10% after three days [34, 35]. Gomez et al (2000) [25] found that at much higher doses (70 μg/ml) after five days of exposure to Cry3Aa protein, it showed moderate toxicity against *P*. *vorax*, supporting that the Cry3A toxin has low activity towards this insect [25]. Binding assays of Cry3Aa, Cry3Ba, Cry3Bb, Cry3Ca and Cry7Aa to brush border membrane vesicles (BBMV) obtained from *P*. *vorax* midgut tissue showed that Cry3Aa, Cry3Bb and Cry3Ca were able to bind to *P*. *vorax* BBMV, while Cry3Ba and Cry7Aa did not [35]. These data suggest that certain Cry toxins have receptors in *P vorax*. Finally, bioassays performed with protoxin proteins from some Cry3Aa mutants showed that mutants located in loops 1 and 3 of domain II (Cry3AaD354E mutant, and the triple Cry3AaR345A-ΔY350-ΔY351 mutant: both located in loop 1 and the triple Cry3AaQ482A-S484A-R485A mutant located in loop 3), showed reduced mortality suggesting that loops 1 and 3 of domain II may be involved in the binding interaction of Cry3Aa to the BBMV from *P*. *vorax* [36]. For these reasons, identification of putative receptor genes of Cry proteins by transcriptome sequencing analysis would be useful for future studies, regarding biocontrol of this important potato crop pest.

We used an RNA-seq approach to characterize the transcriptome of *P*. *vorax*. Total RNA was processed and sequenced using the Illumina technology. The quality control evaluation for the sequencing data was performed using FASTQC and the reconstruction and annotation of the transcriptome was achieved with the Trinity and Trinotate pipelines respectively. An assembly of 517,235 reconstructed transcripts, bearing 232,969 predicted genes (≥298 bp) was obtained, with an average transcript length of 477.8 bp. From the predicted transcripts, we found 870,310 ORFs with a BUSCO completeness of 94.1%. The predicted proteins were analyzed for functional annotation as described in Material and Methods section. With the annotation results, we filtered and clustered the ORFs retaining those with enough evidence of functional annotation and grouping them in clusters with at least 50% of amino acid identity level. (Material and Methods). After filtering, we found a total of 25,631 unigenes with an average mean size of 978 bp and 32,735 proteins. The statistics for the Trinity filtered version are depicted in Table 1.

**Fig 2:**
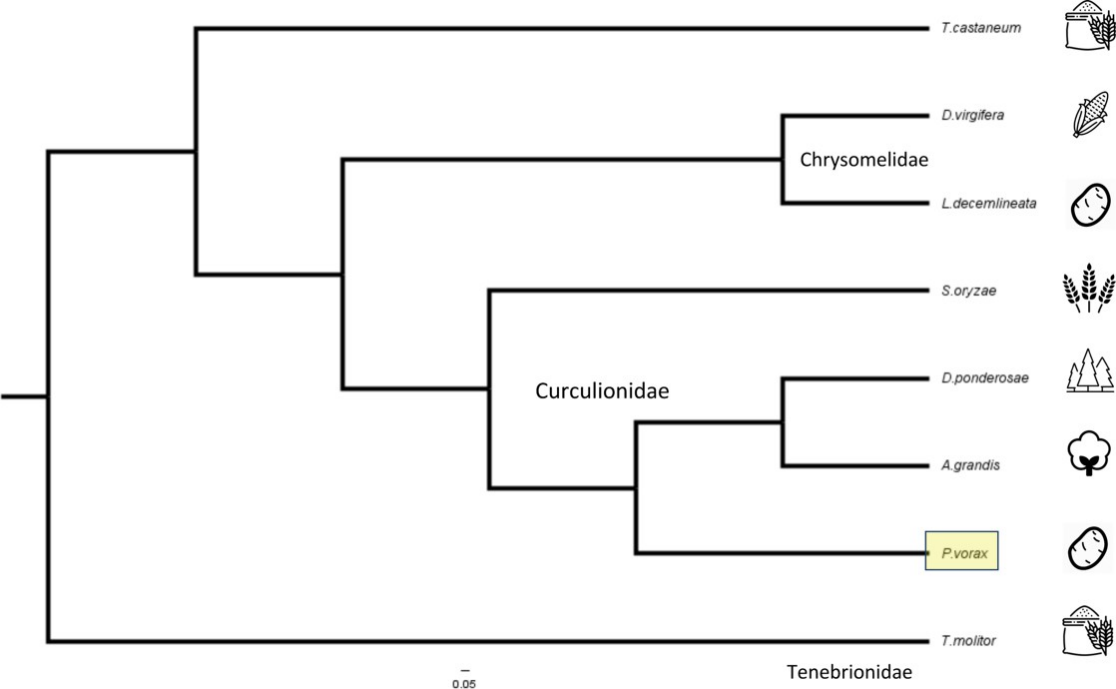
Comparative phylogenetic genomics analysis using 206 orthologous proteins from the *P*. *vorax* transcriptome and other pest beetles.

With the filtered proteins, we performed and ortholog search against all predicted proteins from nine species from the Curculionidae family, with genomes available at the NCBI database and with parasitic relevance. We performed a comparative genomics analysis using 206 selected orthologous protein groups (see Material and methods) present in all species to determine how distant were *P*, *vorax* and other beetles with genomic information in order to find probable Cry-binding protein targets. In Fig 2, a clade for the Curculionidae family can be observed where *P*. *vorax*, *A*. *grandis*, *D*. *ponderosae* and *S*. *oryzae* are part of the same clade as expected from their taxonomy description. The nearest family to them was the Chrysomelidae and the Tenebrionidae family members (*T*. *castaneum* and *T*. *molitor*) acted as an outgroup.

To validate and identify putative homologous genes of Cry-binding proteins such as CAD, APN, ALP, as well as different ABC transporters, we performed a manual inspection the *P*.*vorax* proteins with a BLAST search results with the mentioned functional annotation. A putative CAD gene (TRINITY_DN97354_c5_g1_i3.p1) was found in the transcriptome data of *P*. *vorax* (Table 2). Similarly, APN, and ALPs putative proteins (TRINITY_DN90062_c0_g1_i16.p1 and TRINITY_DN95723_c2_g2_i13.p1, respectively) were identified. Finally different ABC transporters such as ABCC2, ABCG1 and ABCB1 were also identified (TRINITY_DN95792_c0_g1_i7.p1, TRINITY_DN86437_c0_g1_i16.p1 and TRINITY_DN90881_c0_g1_i10.p1, respectively). Their corresponding amino acid percentage of identity and BLAST scores are reported in Table 2. Overall, the sequence with highest identity to the previously reported Cry-binding proteins was ABCB1 with an identity of 62% and the sequence with lowest identity corresponded to ABCG1 with 27% value. Results from the BLAST analysis of all putative *cry* toxin receptors in the orthologous sequences in other annotated coleopteran genomes are shown in Supplementary S1 to S6 Tables. Information in these supplementary tables clearly show that the closest homologs to the cadherin, *APN*, *ABCB1* and *ABCC2* genes of *P*. *vorax* are those from *D*. *ponderosae*; for the *ALP* gene is from *S*. *oryzae*; and for the *ABCG1* gene is from *A*. *grandis*. These data agree with the phylogenetic analysis shown in Fig 2.

**Table 2:** Candidate of Cry toxin-receptor genes in *P*. *vorax* through homology. Reported receptor genes for Cry toxins were used to search for homologs by BLAST in the filtered *P*. *vorax* peptide annotation. For each candidate gene, orthologs were identified in the annotated assemblies of the coleopteran species.

Protein	Species	GenBank ID	*P*. *vorax* ID	*P*. *vorax* length aa	BLAST bit Score	BLAST % identity	BLAST e-value	Orthologs ^a^
**Cadherin**	*Tenebrio molitor*	CAH1373803.1	TRINITY_DN97354_c5_g1_i3.p1	1704	533	32	8.20E-152	Tc_a, Tc_f, Dp_a, Dp_f, Tm, Ld, So, Dv
**ALP**	*Manduca sexta*	XP_030033710.2	TRINITY_DN95723_c2_g2_i13.p1	534	313	39	4.00E-86	Tc_f, Dp_a, Dp_f, Tm, So, Ag, Dv
**APN2**	*Plutella xylostella*	NP_001296022.1	TRINITY_DN90062_c0_g1_i16.p1	951	409	30	1.00E-114	Tc_a, Dp_f, Tm, Ld, So, Dv
**ABCC2**	*Spodoptera frugiperda*	AUO38740.1	TRINITY_DN95792_c0_g1_i7.p1	1346	1064	43	0	Tc_f, Dp_f, Tm, Ld, So, Ag, Dv
**ABCG1**	*Plutella xylostella*	AJQ21779.1	TRINITY_DN86437_c0_g1_i16.p1	636	223	27	6.00E-59	Tc_a, Tc_f, Dp_a, Dp_f, Tm, So, Ag, Dv
**ABCB1**	*Diabrotica virgifera*	XP_050514648.1	TRINITY_DN90881_c0_g1_i10.p1	1124	1443	62	5.00E-88	Tc_a, Tc_f, Dp_a, Dp_f, Tm, Ld, So, Ag, Dv

^a^ Orthologous species are abbreviated as: *Anthonomus grandis*, Ag; *Dendroctonus ponderosae*, Dp; *Diabrotica virgifera*, Dv; *Leptinotarsa decemlineata*, Ld; *Sitophilus oryzae*, So; *Tenebrio molitor*, Tm; *Tribolium castaneum*, Tc.

We then searched for orthologous sequences in other annotated coleopteran genomes as shown also in Table 2, and performed phylogenetic analysis with these data. The resulting phylogenetic trees showed that CAD and APN sequences of *P*. *vorax* were clustered in the same branch with CAD and APN proteins derived from *D*. *ponderosae* indicating higher sequence identity with those proteins. *D*. *ponderosae* also known as the mountain pine beetle is a species of bark beetle native to western conifer forests found from Mexico up to central British Columbia USA. The principal method of control of this pest is with highly toxic chemical insecticides such as pyrethroids. CAD is a potential receptor in Coleopteran larvae since it was demonstrated that in *D*. *virgifera* and *T*. *molitor* the CAD binds to activated Cry3Aa and Cry3Bb [15, 16, 37].

**Fig 3:**
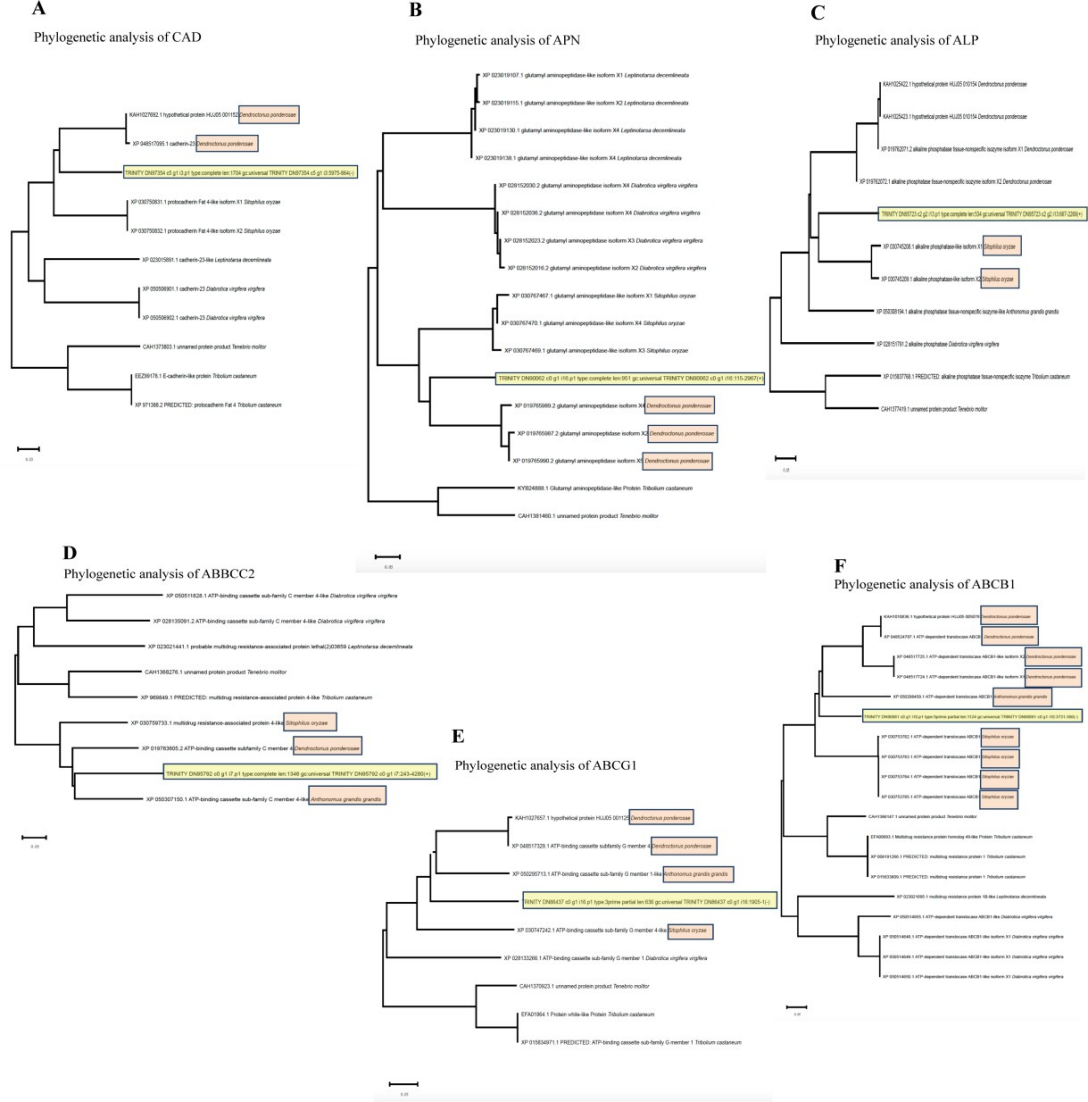
Phylogenetic trees showing the relationship of CAD, APN2, ALP, ABCC2, ABCG1 and ABCB1 sequences from *P*. *vorax* with sequences from other coleopteran insects.

The ALP protein from *P*. *vorax* was clustered in the same branch with ALPs isoforms from *S*. *oryzae* (Fig 3, Panels A, B and C). *S*. *oryzae* is a weevil that belongs to the Sitophilus genus. They are pests of stored grains, such as nut, or seed like rice, wheat, and maize. Freezing infected food below -17°C for three days, or heating food to 60°C for 15 min helps to kill the insects. In addition, it was described that *S*. *oryzae* is susceptible to Cry3Aa toxin [38]. ALP could also be a suitable receptor of Cry toxins in Coleopteran since it was shown that Cry1Ba toxin binds to *A*. *grandis* ALP, while Cry3Aa binds to *T*. *molitor* ALP [39, 40].

Finally, in relation with ABC transporter proteins in all cases these proteins from *P*. *vorax* were clustered with corresponding proteins from *S*. *oryzae*, *D*. *ponderosae* and *A*. *grandis* (Fig 3, Panels D, E and F). *A*. *grandis* also known as boll weevil is a beetle that feeds on cotton buds and flowers. This is a mayor cotton pest in North and South America. Different chemical insecticides have been used for its control such as Calcium arsenate, dichloro-diphenyl-trichloroethane (DDT) and organophosphates. This insect pest is susceptible to several Cry proteins such as Cry1Ia, Cry8Ka, Cry10Aa and Cry22Aa [41–44]. ABC transporters as well as APN and CAD have been identified as binding proteins form Cry8Ea in *H*. *parallela* and *H*. *oblita* [45, 46].

## Conclusions

The reconstructed transcriptome of *P*. *vorax* from larvae RNA, is currently the most complete genetic compendium, representing 94.1% of the expected genomic information for this species. The phylogenetic results obtained with the different putative Cry-receptors in *P*. *vorax* were consistent with the topology of the tree obtained from genomics analysis as shown in Fig 2, suggesting that the relationship between the probable Cry-binding proteins in *P*. *vorax* and other pest beetles is reliable, and they are good Cry target candidates. However, the experimental characterization and validation of these targets is needed to confirm that the proteins are present and their predicted functions can result in the interaction with different Cry toxins.

To our knowledge, this study represent a substantial and valuable novel transcriptomic data from *P*.*vorax* and identified homologous proteins to the reported Cry toxin receptors identified in other insect pests that would be useful for future functional studies and to overcome potential problems of insect resistance to Cry toxin insecticides. The information described in this study will be important knowledge for future research performed with this important potato pest.

## Supporting information

S1 Table BLAST results for TRINITY_DN97354_c5_g1_i3.p1 with cadherin orthologs. (DOCX)

S2 Table BLAST results for TRINITY_DN95723_c2_g2_i13.p1 with ALP orthologs. (DOCX)

S3 Table BLAST results for TRINITY_DN90062_c0_g1_i16.p1 with APN2 orthologs. (DOCX)

S4 Table BLAST results for TRINITY_DN90881_c0_g1_i10.p1 with ABCB1 orthologs. (DOCX)

S5 Table BLAST results for TRINITY_DN95792_c0_g1_i7.p1 with ABCC2 orthologs. (DOCX)

S6 Table BLAST results for TRINITY_DN86437_c0_g1_i16.p1 with ABCG1 orthologs. (DOCX)
