# Supplementary material for: Identification of Cry toxin receptor genes homologs in a de novo transcriptome of Premnotrypes vorax (Coleoptera: Curculionidae)
Source: PLoS One. 2023 Sep 14;18(9):e0291546. doi: 10.1371/journal.pone.0291546 (PMC10501650; doi:10.1371/journal.pone.0291546)
Supplement: S1 Table — (DOCX) [file pone.0291546.s001.docx]

Supporting Information

**S1 Table.** BLAST results for TRINITY_DN97354_c5_g1_i3.p1 with cadherin orthologs.

| **Subject** | **Identity** | **Coverage** | **Score** | **E-Value** | **Subject Annotation** |  |
| --- | --- | --- | --- | --- | --- | --- |
| XP_048517095.1 | 52.7473 | 97.8873 | 4353 | 0 | cadherin-23 [*Dendroctonus ponderosae*] | |
| KAH1027692.1 | 52.2086 | 89.6127 | 4072 | 0 | hypothetical protein HUJ05_001152 [*Dendroctonus ponderosae*] | |
| XP_030750832.1 | 51.4002 | 97.5939 | 4071 | 0 | protocadherin Fat 4-like isoform X2 [*Sitophilus oryzae*] | |
| XP_030750831.1 | 51.3986 | 97.5939 | 4066 | 0 | protocadherin Fat 4-like isoform X1 [*Sitophilus oryzae*] | |
| XP_023015891.1 | 38.2578 | 94.0141 | 2523 | 0 | cadherin-23-like [*Leptinotarsa decemlineata*] | |
| XP_050506901.1 | 36.9151 | 95.8333 | 2345 | 0 | cadherin-23 [*Diabrotica virgifera virgifera*] | |
| XP_050506902.1 | 36.9151 | 95.8333 | 2345 | 0 | cadherin-23 [*Diabrotica virgifera virgifera*] | |
| EEZ99178.1 | 33.494 | 87.1479 | 1745 | 0 | E-cadherin-like protein [*Tribolium castaneum*] | |
| XP_971388.2 | 33.494 | 87.1479 | 1745 | 0 | PREDICTED: protocadherin Fat 4 [*Tribolium castaneum*] | |
| CAH1373803.1 | 32.5378 | 89.3192 | 1650 | 0 | unnamed protein product [*Tenebrio molitor*] | |
