# Supplementary material for: Identification of Cry toxin receptor genes homologs in a de novo transcriptome of Premnotrypes vorax (Coleoptera: Curculionidae)
Source: PLoS One. 2023 Sep 14;18(9):e0291546. doi: 10.1371/journal.pone.0291546 (PMC10501650; doi:10.1371/journal.pone.0291546)
Supplement: S2 Table — (DOCX) [file pone.0291546.s002.docx]

Supporting Information **S2 Table.** BLAST results for TRINITY_DN95723_c2_g2_i13.p1 with ALP orthologs.

| **Subject** | **Identity** | **Coverage** | **Score** | **E-Value** | **Subject Annotation** |
| --- | --- | --- | --- | --- | --- |
| XP_030745208.1 | 74.5318 | 98.1273 | 2118 | 0 | alkaline phosphatase-like isoform X1 [*Sitophilus oryzae*] |
| XP_030745209.1 | 76.4133 | 94.382 | 2115 | 0 | alkaline phosphatase-like isoform X2 [*Sitophilus oryzae*] |
| XP_019762071.2 | 73.3333 | 96.6292 | 2104 | 0 | alkaline phosphatase, tissue-nonspecific isozyme isoform X1 [*Dendroctonus ponderosae*] |
| XP_050308194.1 | 73.5632 | 96.4419 | 2050 | 0 | alkaline phosphatase, tissue-nonspecific isozyme-like [*Anthonomus grandis grandis*] |
| XP_019762072.1 | 77.9956 | 85.7678 | 1999 | 0 | alkaline phosphatase, tissue-nonspecific isozyme isoform X2 [*Dendroctonus ponderosae*] |
| KAH1025423.1 | 68 | 90.4494 | 1907 | 0 | hypothetical protein HUJ05_010154 [*Dendroctonus ponderosae*] |
| KAH1025422.1 | 68 | 90.4494 | 1907 | 0 | hypothetical protein HUJ05_010154 [*Dendroctonus ponderosae*] |
| XP_028151791.2 | 65.3768 | 91.3858 | 1766 | 0 | alkaline phosphatase [*Diabrotica virgifera virgifera*] |
| CAH1377419.1 | 58.3174 | 95.1311 | 1623 | 0 | unnamed protein product [*Tenebrio molitor*] |
| XP_015837768.1 | 60.7362 | 90.824 | 1596 | 0 | PREDICTED: alkaline phosphatase, tissue-nonspecific isozyme [*Tribolium castaneum*] |
