# Supplementary material for: Identification of Cry toxin receptor genes homologs in a de novo transcriptome of Premnotrypes vorax (Coleoptera: Curculionidae)
Source: PLoS One. 2023 Sep 14;18(9):e0291546. doi: 10.1371/journal.pone.0291546 (PMC10501650; doi:10.1371/journal.pone.0291546)
Supplement: S3 Table — (DOCX) [file pone.0291546.s003.docx]

Supporting Information

**S3 Table.** BLAST results for TRINITY_DN90062_c0_g1_i16.p1 with APN2 orthologs.

| **Subject** | **Identity** | **Coverage** | **Score** | **E-Value** | **Subject Annotation** |
| --- | --- | --- | --- | --- | --- |
| XP_019765989.2 | 74.078 | 98.4227 | 3801 | 0 | glutamyl aminopeptidase isoform X4 [*Dendroctonus ponderosae*] |
| XP_019765990.2 | 76.8614 | 91.7981 | 3675 | 0 | glutamyl aminopeptidase isoform X5 [*Dendroctonus ponderosae*] |
| XP_019765987.2 | 76.8614 | 91.7981 | 3671 | 0 | glutamyl aminopeptidase isoform X2 [*Dendroctonus ponderosae*] |
| XP_030767469.1 | 72.2105 | 98.633 | 3644 | 0 | glutamyl aminopeptidase-like isoform X3 [*Sitophilus oryzae*] |
| XP_030767467.1 | 75.0288 | 91.2723 | 3513 | 0 | glutamyl aminopeptidase-like isoform X1 [*Sitophilus oryzae*] |
| XP_030767470.1 | 75.0288 | 91.2723 | 3511 | 0 | glutamyl aminopeptidase-like isoform X4 [*Sitophilus oryzae*] |
| XP_023019115.1 | 62.3656 | 94.5321 | 3076 | 0 | glutamyl aminopeptidase-like isoform X2 [*Leptinotarsa decemlineata*] |
| XP_023019107.1 | 62.3656 | 94.5321 | 3073 | 0 | glutamyl aminopeptidase-like isoform X1 [*Leptinotarsa decemlineata*] |
| XP_023019138.1 | 65.2425 | 90.326 | 3055 | 0 | glutamyl aminopeptidase-like isoform X4 [*Leptinotarsa decemlineata*] |
| XP_023019130.1 | 65.2425 | 90.326 | 3055 | 0 | glutamyl aminopeptidase-like isoform X4 [*Leptinotarsa decemlineata*] |
| XP_028152016.2 | 64.0878 | 90.326 | 2965 | 0 | glutamyl aminopeptidase isoform X2 [*Diabrotica virgifera virgifera*] |
| XP_028152023.2 | 64.0878 | 90.326 | 2965 | 0 | glutamyl aminopeptidase isoform X3 [*Diabrotica virgifera virgifera*] |
| XP_028152036.2 | 64.0878 | 90.326 | 2964 | 0 | glutamyl aminopeptidase isoform X4 [*Diabrotica virgifera virgifera*] |
| XP_028152030.2 | 64.0878 | 90.326 | 2964 | 0 | glutamyl aminopeptidase isoform X4 [*Diabrotica virgifera virgifera*] |
| CAH1381460.1 | 57.957 | 96.3197 | 2830 | 0 | unnamed protein product [*Tenebrio molitor*] |
| KYB24888.1 | 56.0166 | 95.0578 | 2690 | 0 | Glutamyl aminopeptidase-like Protein [*Tribolium castaneum*] |
