# Supplementary material for: Identification of Cry toxin receptor genes homologs in a de novo transcriptome of Premnotrypes vorax (Coleoptera: Curculionidae)
Source: PLoS One. 2023 Sep 14;18(9):e0291546. doi: 10.1371/journal.pone.0291546 (PMC10501650; doi:10.1371/journal.pone.0291546)
Supplement: S4 Table — (DOCX) [file pone.0291546.s004.docx]

Supporting Information

**S4 Table.** BLAST results for TRINITY_DN90881_c0_g1_i10.p1with ABCB1 orthologs.

| **Subject** | **Identity** | **Coverage** | **Score** | **E-Value** | **Subject Annotation** |
| --- | --- | --- | --- | --- | --- |
| XP_048524797.1 | 76.6696 | 99.5552 | 4692 | 0 | ATP-dependent translocase ABCB1 [*Dendroctonus ponderosae*] |
| XP_030753785.1 | 75.5752 | 99.4662 | 4600 | 0 | ATP-dependent translocase ABCB1 [*Sitophilus oryzae*] |
| XP_030753784.1 | 75.5752 | 99.4662 | 4600 | 0 | ATP-dependent translocase ABCB1 [*Sitophilus oryzae*] |
| XP_030753783.1 | 75.5752 | 99.4662 | 4600 | 0 | ATP-dependent translocase ABCB1 [*Sitophilus oryzae*] |
| XP_030753782.1 | 75.5752 | 99.4662 | 4600 | 0 | ATP-dependent translocase ABCB1 [*Sitophilus oryzae*] |
| KAH1010836.1 | 75.6901 | 98.3986 | 4598 | 0 | hypothetical protein HUJ05_005078 [*Dendroctonus ponderosae*] |
| XP_048517725.1 | 74.9777 | 99.3772 | 4580 | 0 | ATP-dependent translocase ABCB1-like isoform X2 [*Dendroctonus ponderosae*] |
| XP_048517724.1 | 73.8596 | 99.4662 | 4572 | 0 | ATP-dependent translocase ABCB1-like isoform X1 [*Dendroctonus ponderosae*] |
| XP_050298459.1 | 73.9556 | 99.7331 | 4521 | 0 | ATP-dependent translocase ABCB1 [*Anthonomus grandis grandis*] |
| CAH1366147.1 | 65.5536 | 98.2206 | 3959 | 0 | unnamed protein product [*Tenebrio molitor*] |
| XP_015833809.1 | 64.1026 | 99.1103 | 3918 | 0 | PREDICTED: multidrug resistance protein 1 [*Tribolium castaneum*] |
| XP_008191266.1 | 64.1026 | 99.1103 | 3918 | 0 | PREDICTED: multidrug resistance protein 1 [*Tribolium castaneum*] |
| EFA00893.1 | 64.1026 | 99.1103 | 3918 | 0 | Multidrug resistance protein homolog 49-like Protein [*Tribolium castaneum*] |
| XP_050514648.1 | 62.1598 | 97.9537 | 3822 | 0 | ATP-dependent translocase ABCB1-like isoform X1 [*Diabrotica virgifera virgifera]* |
| XP_050514649.1 | 62.1598 | 97.9537 | 3822 | 0 | ATP-dependent translocase ABCB1-like isoform X1 [*Diabrotica virgifera virgifera*] |
| XP_050514650.1 | 62.1598 | 97.9537 | 3822 | 0 | ATP-dependent translocase ABCB1-like isoform X1 [*Diabrotica virgifera virgifera*] |
| XP_050514955.1 | 60.9778 | 99.1103 | 3702 | 0 | ATP-dependent translocase ABCB1-like [*Diabrotica virgifera virgifera*] |
| XP_023021695.1 | 57.6211 | 94.395 | 3288 | 0 | multidrug resistance protein 1B-like [*Leptinotarsa decemlineata*] |
| XP_030753782.1 | 37.585 | 48.6655 | 941 | 6.83E-111 | ATP-dependent translocase ABCB1 [*Sitophilus oryzae*] |
| XP_030753783.1 | 37.585 | 48.6655 | 941 | 6.83E-111 | ATP-dependent translocase ABCB1 [*Sitophilus oryzae*] |
| XP_030753784.1 | 37.585 | 48.6655 | 941 | 6.83E-111 | ATP-dependent translocase ABCB1 [*Sitophilus oryzae*] |
| XP_030753785.1 | 37.585 | 48.6655 | 941 | 6.83E-111 | ATP-dependent translocase ABCB1 [*Sitophilus oryzae*] |
| XP_015833809.1 | 35.7576 | 52.4911 | 913 | 5.91E-107 | PREDICTED: multidrug resistance protein 1 [*Tribolium castaneum*] |
| XP_008191266.1 | 35.7576 | 52.4911 | 913 | 5.91E-107 | PREDICTED: multidrug resistance protein 1 [*Tribolium castaneum*] |
| EFA00893.1 | 35.7576 | 52.4911 | 913 | 5.91E-107 | Multidrug resistance protein homolog 49-like Protein [*Tribolium castaneum*] |
| XP_048524797.1 | 35.2273 | 51.4235 | 912 | 9.39E-107 | ATP-dependent translocase ABCB1 [*Dendroctonus ponderosae*] |
| CAH1366147.1 | 37.1859 | 49.2883 | 894 | 2.43E-104 | unnamed protein product [*Tenebrio molitor*] |
| KAH1010836.1 | 34.9026 | 50.5338 | 892 | 3.42E-104 | hypothetical protein HUJ05_005078 [*Dendroctonus ponderosae*] |
| XP_048517724.1 | 35.8621 | 47.5979 | 868 | 9.70E-101 | ATP-dependent translocase ABCB1-like isoform X1 [*Dendroctonus ponderosae*] |
| XP_048517725.1 | 35.8621 | 47.5979 | 866 | 1.43E-100 | ATP-dependent translocase ABCB1-like isoform X2 [*Dendroctonus ponderosae*] |
| XP_050514955.1 | 37.3311 | 49.2883 | 864 | 2.58E-100 | ATP-dependent translocase ABCB1-like [*Diabrotica virgifera virgifera*] |
| XP_050514648.1 | 35.473 | 49.2883 | 844 | 1.30E-97 | ATP-dependent translocase ABCB1-like isoform X1 [*Diabrotica virgifera virgifera*] |
| XP_050514649.1 | 35.473 | 49.2883 | 844 | 1.30E-97 | ATP-dependent translocase ABCB1-like isoform X1 [*Diabrotica virgifera virgifera*] |
| XP_050514650.1 | 35.473 | 49.2883 | 844 | 1.30E-97 | ATP-dependent translocase ABCB1-like isoform X1 [*Diabrotica virgifera virgifera*] |
| XP_050298459.1 | 35.533 | 48.8434 | 839 | 6.12E-97 | ATP-dependent translocase ABCB1 [*Anthonomus grandis grandis*] |
| XP_023021695.1 | 36.8512 | 45.8185 | 742 | 3.03E-84 | multidrug resistance protein 1B-like [*Leptinotarsa decemlineata*] |
