# Supplementary material for: Identification of Cry toxin receptor genes homologs in a de novo transcriptome of Premnotrypes vorax (Coleoptera: Curculionidae)
Source: PLoS One. 2023 Sep 14;18(9):e0291546. doi: 10.1371/journal.pone.0291546 (PMC10501650; doi:10.1371/journal.pone.0291546)
Supplement: S5 Table — (DOCX) [file pone.0291546.s005.docx]

Supporting Information

**S5 Table.** BLAST results for TRINITY_DN95792_c0_g1_i7.p1 with ABCC2 orthologs.

| **Subject** | **Identity** | **Coverage** | **Score** | **E-Value** | **Subject Annotation** |
| --- | --- | --- | --- | --- | --- |
| XP_019763605.2 | 71.4286 | 96.7773 | 3954 | 0 | ATP-binding cassette subfamily C member 4 [*Dendroctonus ponderosae*] |
| XP_030759733.1 | 70.9646 | 96.9727 | 3882 | 0 | multidrug resistance-associated protein 4-like [*Sitophilus oryzae*] |
| XP_050307150.1 | 70.0593 | 95.1172 | 3757 | 0 | ATP-binding cassette sub-family C member 4-like [*Anthonomus grandis grandis*] |
| XP_969849.1 | 54.2406 | 94.9219 | 2923 | 0 | PREDICTED: multidrug resistance-associated protein 4-like [*Tribolium castaneum*] |
| CAH1368276.1 | 55.1793 | 93.6523 | 2884 | 0 | unnamed protein product [*Tenebrio molitor*] |
| XP_028135091.2 | 53.3528 | 98.2422 | 2782 | 0 | ATP-binding cassette sub-family C member 4-like [*Diabrotica virgifera virgifera*] |
| XP_050511828.1 | 52.0428 | 98.1445 | 2742 | 0 | ATP-binding cassette sub-family C member 4-like [*Diabrotica virgifera virgifera*] |
| CAH1368276.1 | 40.1135 | 92.8711 | 1995 | 0 | unnamed protein product [*Tenebrio molitor*] |
| XP_023021441.1 | 53.8231 | 63.6719 | 1881 | 0 | probable multidrug resistance-associated protein lethal(2)03659 [*Leptinotarsa decemlineata*] |
| XP_969849.1 | 23.6641 | 46.6797 | 240 | 2.77E-23 | PREDICTED: multidrug resistance-associated protein 4-like [*Tribolium castaneum*] |
| XP_023021441.1 | 26.2931 | 38.5742 | 217 | 1.29E-20 | probable multidrug resistance-associated protein lethal(2)03659 [*Leptinotarsa decemlineata*] |
| XP_050307150.1 | 29.6875 | 22.9492 | 208 | 1.53E-19 | ATP-binding cassette sub-family C member 4-like [*Anthonomus grandis grandis*] |
| XP_028135091.2 | 24.3187 | 41.9922 | 198 | 2.86E-18 | ATP-binding cassette sub-family C member 4-like [*Diabrotica virgifera virgifera*] |
| CAH1368276.1 | 23.1618 | 46.3867 | 196 | 5.53E-18 | unnamed protein product [*Tenebrio molitor*] |
| XP_050511828.1 | 29.8077 | 17.4805 | 194 | 7.85E-18 | ATP-binding cassette sub-family C member 4-like [*Diabrotica virgifera virgifera*] |
| XP_019763605.2 | 29.6137 | 20.7031 | 182 | 2.00E-16 | ATP-binding cassette subfamily C member 4 [*Dendroctonus ponderosae*] |
| XP_030759733.1 | 30.4721 | 20.7031 | 177 | 7.14E-16 | multidrug resistance-associated protein 4-like [*Sitophilus oryzae*] |
| CAH1368276.1 | 23.5012 | 34.1797 | 166 | 1.60E-14 | unnamed protein product [*Tenebrio molitor*] |
