# Supplementary material for: Identification of Cry toxin receptor genes homologs in a de novo transcriptome of Premnotrypes vorax (Coleoptera: Curculionidae)
Source: PLoS One. 2023 Sep 14;18(9):e0291546. doi: 10.1371/journal.pone.0291546 (PMC10501650; doi:10.1371/journal.pone.0291546)
Supplement: S6 Table — (DOCX) [file pone.0291546.s006.docx]

Supporting Information

**S6 Table.** BLAST results for TRINITY_DN86437_c0_g1_i16.p1 with ABCG1 orthologs.

| **Subject** | **Identity** | **Coverage** | **Score** | **E-Value** | **Subject Annotation** |
| --- | --- | --- | --- | --- | --- |
| XP_050295713.1 | 73.8095 | 98.2677 | 2545 | 0 | ATP-binding cassette sub-family G member 1-like [*Anthonomus grandis grandis]* |
| XP_030747242.1 | 70.5882 | 97.9528 | 2397 | 0 | ATP-binding cassette sub-family G member 4-like [*Sitophilus oryzae*] |
| XP_028133266.1 | 64.0945 | 98.8976 | 2246 | 0 | ATP-binding cassette sub-family G member 1 [*Diabrotica virgifera virgifera*] |
| XP_015834971.1 | 61.129 | 96.063 | 2106 | 0 | PREDICTED: ATP-binding cassette sub-family G member 1 [*Tribolium castaneum*] |
| EFA01864.1 | 61.129 | 96.063 | 2106 | 0 | Protein white-like Protein [*Tribolium castaneum*] |
| CAH1370923.1 | 61.5137 | 97.0079 | 2067 | 0 | unnamed protein product [*Tenebrio molitor*] |
| XP_048517329.1 | 74.4422 | 77.1654 | 1969 | 0 | ATP-binding cassette subfamily G member 4 [*Dendroctonus ponderosae*] |
| KAH1027657.1 | 73.4 | 77.3228 | 1951 | 0 | hypothetical protein HUJ05_001125 [*Dendroctonus ponderosae*] |
